# Glycosylation of TRPM4 and TRPM5 channels: molecular determinants and functional aspects

**DOI:** 10.3389/fncel.2014.00052

**Published:** 2014-02-24

**Authors:** Ninda Syam, Jean-Sébastien Rougier, Hugues Abriel

**Affiliations:** Department of Clinical Research, and Swiss National Centre of Competence in Research (NCCR) TransCure, University of BernBern, Switzerland

**Keywords:** TRPM4, TRPM5, N-linked glycosylation, ion channels, regulation

## Abstract

The transient receptor potential channel, TRPM4, and its closest homolog, TRPM5, are non-selective cation channels that are activated by an increase in intracellular calcium. They are expressed in many cell types, including neurons and myocytes. Although the electrophysiological and pharmacological properties of these two channels have been previously studied, less is known about their regulation, in particular their post-translational modifications. We, and others, have reported that wild-type (WT) TRPM4 channels expressed in HEK293 cells, migrated on SDS-PAGE gel as doublets, similar to other ion channels and membrane proteins. In the present study, we provide evidence that TRPM4 and TRPM5 are each N-linked glycosylated at a unique residue, Asn^992^ and Asn^932^, respectively. N-linked glycosylated TRPM4 is also found in native cardiac cells. Biochemical experiments using HEK293 cells over-expressing WT TRPM4/5 or N992Q/N932Q mutants demonstrated that the abolishment of N-linked glycosylation did not alter the number of channels at the plasma membrane. In parallel, electrophysiological experiments demonstrated a decrease in the current density of both mutant channels, as compared to their respective controls, either due to the Asn to Gln mutations themselves or abolition of glycosylation. To discriminate between these possibilities, HEK293 cells expressing TRPM4 WT were treated with tunicamycin, an inhibitor of glycosylation. In contrast to N-glycosylation signal abolishment by mutagenesis, tunicamycin treatment led to an increase in the TRPM4-mediated current. Altogether, these results demonstrate that TRPM4 and TRPM5 are both N-linked glycosylated at a unique site and also suggest that TRPM4/5 glycosylation seems not to be involved in channel trafficking, but mainly in their functional regulation.

## Introduction

TRPM4, and its closest homolog TRPM5, are members of the transient receptor potential (TRP) ion channel gene family (Clapham et al., 2005; Ramsey et al., 2006). As with all TRP channels, they consist of tetramers of alpha subunits and each monomer contains six transmembrane segments. The selectivity filter is located in the loop connecting the fifth and sixth transmembrane domains. The cytoplasmic N- and C-termini contain a number of potential protein-protein interaction domains (Nilius et al., 2005b). TRPM4 and TRPM5 channels are both activated by an increase in intracellular calcium and are only permeable to monovalent cations, such as sodium and potassium. In humans, TRPM4 is expressed in many cell types (Nilius et al., 2004). The human TRPM4b long splice variant is the only form that has been shown to mediate ionic current in heterologous expression systems, whereas the role of the short splice TRPM4a variant remains unknown (Murakami et al., 2003; Nilius et al., 2004). In the present study, we investigated the functional splice variant TRPM4b (subsequently referred to as TRPM4) and its closest homolog TRPM5.

Thus, far, the roles of TRPM4 have mainly been studied in immune cells (Launay et al., 2004; Barbet et al., 2008; Shimizu et al., 2009), pancreatic beta cells (Cheng et al., 2007), arterial and venous smooth muscle cells (Gonzales et al., 2010; Simard et al., 2010), skeletal muscle cells (Kruger et al., 2008), adrenal gland chromaffin cells (Mathar et al., 2010), cardiac cells (Guinamard et al., 2006a,b; Demion et al., 2007; Guinamard, 2007; Mathar et al., 2013), and neuronal cells (Schattling et al., 2012; Kim et al., 2013). TRPM5 has been shown to play an important role in taste sensory cells (Zhang et al., 2007) and in neurons (Egorov et al., 2002, 2006; Teruyama et al., 2011). The importance of TRPM4 in disease has been underlined by the observation that many pathogenic genetic variants have been found in patients with cardiac conduction disorders (Abriel et al., 2012), and that TRPM4 knock-out mice develop less neurological alterations in a mouse model of multiple sclerosis (Schattling et al., 2012).

A growing number of studies show that post-translational modification of TRPM4, such as phosphorylation by PKC (Earley et al., 2007; Crnich et al., 2010) and SUMOylation, play a role in regulating their density at the plasma membrane (Kruse et al., 2009; Liu et al., 2010). In addition, TRPM4 Western blots show that it migrates in SDS-PAGE gels as two distinct bands, suggesting that it may be glycosylated (Amarouch et al., 2013; Woo et al., 2013b). Thus, far, the molecular determinants and the function of this glycosylation are only partially understood.

In this work, we used HEK293 cells transiently transfected with either TRPM4 or TRPM5 to investigate whether these channel proteins are glycosylated, and to determine the functional consequences of this post-translational modification. Biochemical and pharmacological experiments using mutant forms of TRPM4/5 channels revealed a unique N-glycosylation site close to the selectivity filter. Cell surface biotinylation experiments and patch-clamp recording in whole cell configuration suggest that TRPM4 and TRPM5 glycosylation plays a role in their function as channel proteins rather than for their expression at the plasma membrane. The results of the present study confirm and expand previous results, but at the same time highlight several differences to a recent study by Woo et al. (2013a).

## Experimental procedures

### Plasmids

Two plasmids, human HA-TRPM4 WT (GenBank AF497623; Uniprot Q8TD43-1) and TRPM5 WT (GenBank NM_014555.3; Uniprot Q9NZQ8-1), were received from Dr. Bouvagnet (University of Lyon, France) and from Dr. Vennekens (University of Leuven, Belgium), respectively. The constructs were mutagenized using Quickchange II XL (Agilent, Santa Clara, California, USA), according to the manufacturer's protocol. The primer sequences (5′ to 3′) used for mutagenesis were as follows: S TRPM4 a2974c_c2976g TCATGGAGCACAGCCAGTGCTCGTCGGAGCC, AS TRPM4 a2974c_c2976g GGCTCCGACGAGCACTGGCTGTGCTCCATGA, S TRPM5 a2794c_c2796g ATGAAGCCCGTGTGCAGTGCTCCACCCACCC, AS TRPM5 a2794c_c2796g GGGTGGGTGGAGCACTGCACACGGGCTTCAT.

### Transfections

For biochemical experiments, HEK293 cells were individually transiently transfected with the following plasmids: 240 ng of HA-TRPM4 WT or HA-TRPM4 N992Q, 2 μg of either TRPM5 WT or TRPM5 N932Q in a P100 dish (BD Falcon, Durham, North Carolina, USA), mixed with 30 μ l of JetPEI (Polyplus transfection, Illkirch, France) and 250 μ l of 150 mM NaCl. The cells were incubated for 48 h at 37°C with 5% CO_2_. For electrophysiological studies, T25 cm^2^ flasks of HEK293 cells were transiently co-transfected using X-tremeGENE 9 DNA transfection mix reagent (Roche Diagnostics, IN, USA) with 0.3 μg of WT, mutant TRPM4, or TRPM5 channels. These transfections included 0.2 μg of cDNA encoding CD8 antigen as a reporter gene. Anti-CD8 beads(Dynal, Oslo, Norway) were used to identify transfected cells, and only CD8-displaying cells were analyzed.Cells were used 48 h after transfection.

### Cell surface biotinylation assays

Following 48 h of incubation, transiently transfected HEK293 cells previously washed twice with cold 1x PBS were treated with 0.5 mg/ml EZ-link™ Sulfo-NHS-SS-Biotin (Thermo Scientific, Rockford, Illinois, USA) in cold 1X PBS for 15 min at 4°C. Subsequently, the cells were washed twice with 200 mM Glycine in cold 1X PBS and twice with cold 1X PBS to inactivate and remove the excess biotin, respectively. The cells were then lysed with 1X lysis buffer [50 mM HEPES pH 7.4; 150 mM NaCl; 1.5 mM MgCl_2_; 1 mM EGTA pH 8; 10% Glycerol; 1% Triton X-100; 1X Complete Protease Inhibitor Cocktail (Roche, Mannheim, Germany)] for 1 h at 4°C. Cell lysates were centrifuged at 16,000 g, 4°C for 15 min. Two milligrams of the supernatant were incubated with 50 μl Streptavidin Sepharose High Performance beads (GE Healthcare, Uppsala, Sweden) for 2 h at 4°C, and the remaining supernatant was kept as the input. The beads were subsequently washed five times with 1X lysis buffer before elution with 50 μl of 2X NuPAGE sample buffer (Invitrogen, Carlsbad, California, USA) plus 100 mM DTT at 37°C for 30 min. These biotinylated fractions were analyzed as TRPM4 expressed at the cell surface. The input fractions, analyzed as total expression of TRPM4, were resuspended with 4X NuPAGE Sample Buffer plus 100 mM DTT, yielding a concentration of 1 mg/ml, and then incubated at 37°C for 30 min.

### Deglycosylation assays

Deglycosylation with PNGase F and Endo H. HEK293 cells or homogenized human atrial samples were lysed with 1X lysis buffer [50 mM HEPES pH 7.4; 150 mM NaCl; 1.5 mM MgCl_2_; 1 mM EGTA pH 8.0; 10% Glycerol; 1% Triton X-100; 1X Complete Protease Inhibitor Cocktail (Roche, Mannheim, Germany)] for 1 h at 4°C. The lysates were then centrifuged at 16,000 g, 4°C for 15 min. Forty micrograms of these lysates were denatured for 30 min at 37°C in the presence of 1X Glycoprotein denaturing buffer and bidistilled H_2_O in a total volume of 21 μl. Following this incubation, glycosylation mix was added [PNGase F mix: 1X G7 buffer, 1X NP40 and 1500 units PNGase F; Endo H mix: 1X G5 buffer and 3000 units Endo H (New England Biolabs, Ipswich, MA, USA)] to the denatured samples and incubated for 1 h at 37°C. To stop the reaction, 10 μl of 4X NuPAGE sample buffer (Invitrogen, Carlsbad, California, USA) plus 100 mM DTT were added to the deglycosylated samples. For deglycosylation on biotinylated samples, denaturation was performed after the 2 h incubation of whole cell lysates with streptavidin beads. After aspirating the lysate, 35 μl of bidistillated H_2_O and 5 μl of 10X Glycoprotein denaturing buffer were added prior to denaturation at 37°C for 30 min. Following this incubation, the glycosylation mix (PNGase F mix: 6 μl 10X G7 buffer, 6 μl 10X NP40 and 2000 units PNGase F or Endo H mix: 6 μl 10X G5 buffer and 4000 units Endo H) was added to the denatured samples and incubated for 1 h at 37°C. The samples were subsequently washed five times with the same lysis buffer (800 g, 4°C, 2 min) before elution with 50 μl of 2X NuPAGE sample buffer plus 100 mM DTT at 37°C for 30 min. Human tissue acquisition was performed in accordance with the Institutional Review Board and individual patient consent was obtained.

### Western blot

Protein samples were analyzed on 9% polyacrylamide gels, transferred with the TurboBlot dry blot system (Biorad, Hercules, CA, USA) and detected with anti-TRPM4 (generated by Pineda, Berlin, Germany), anti-TRPM5 ab154788 (Abcam, Cambridge, UK), anti-Na^+^/K^+^ ATPase α1 ab7671 (Abcam, Cambridge, UK) and anti α-actin A2066 (Sigma, St. Louis, Missouri, USA) antibodies using SNAP i.d. (Millipore, Billerica, MA, USA). The anti-TRPM4 antibody was generated by Pineda (Berlin, Germany) using the following peptide sequence: NH2-CRDKRESDSERLKRTSQKV-CONH2. A fraction of the antisera, which was subsequently used in this study, was then affinity purified.

### Cellular electrophysiology

Whole-cell currents were measured at room temperature (22–23°C) using a VE-2 (Alembic Instruments) amplifier. Pipette solution contained (in mM): 100 CsAspartate, 20 CsCl, 4 Na_2_ATP, 1 MgCl_2_, and 10 HEPES. The pH was adjusted to 7.2 with CsOH, and the Ca^2+^ free concentration ranged to 100 μM calculated by WEBMAXCLITE program (http://www.stanford.edu/~cpatton/downloads.htm). Bath solutions contained (in mM): 156 NaCl, 1.5 CaCl_2_, 1 MgCl_2_, 6 CsCl 10 D-glucose, and 10 HEPES. The pH was adjusted to 7.4 with NaOH. Data were analyzed using pClampsoftware, version 10.2 (Axon Instruments, Union City, California, USA).

### Drugs

Tunicamycin was purchased from Sigma-Aldrich (Sigma-Aldrich, Dorset, United Kingdom) and was dissolved in DMSO. DMSO did not exceed 0.1% in the final solution.

### Statistical analysis

Data are presented as mean ± s.e.m. Unpaired, two-tailed Student's *t*-test was used to compare the means; *p* < 0.05 was considered significant.

## Results

### TRPM4 channel is N-linked glycosylated at Asn^992^

**Figure 1:**
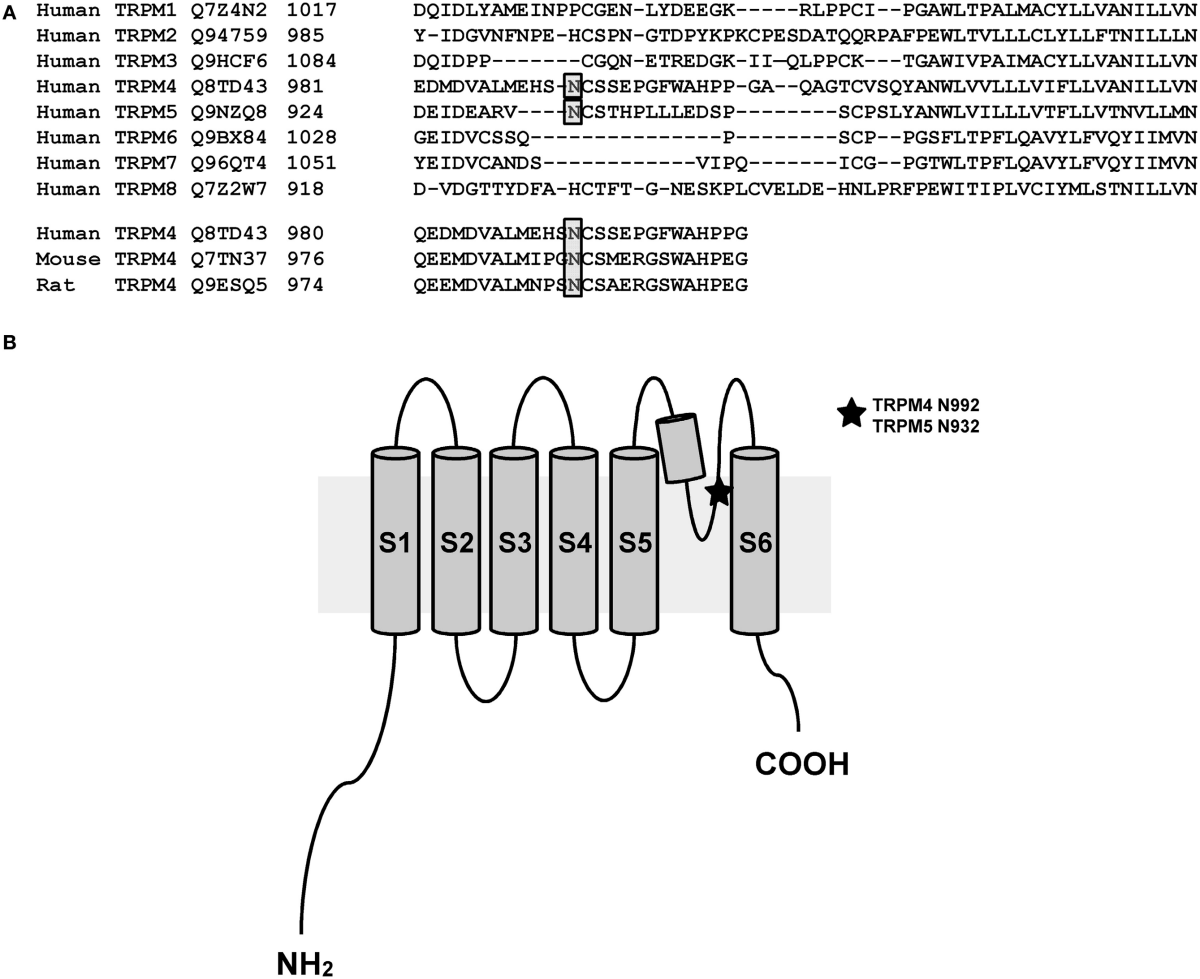
**N-glycosylation site of TRPM4 and TRPM5. (A)** A bioinformatic search reveals a putative site for N-linked glycosylation in TRPM4 that is conserved in different organisms and the closely related TRPM5. **(B)** Illustration showing the location of N-linked glycosylation site in the pore-forming region between transmembrane segments 5 and 6 of TRPM4 and TRPM5.

**Figure 2:**
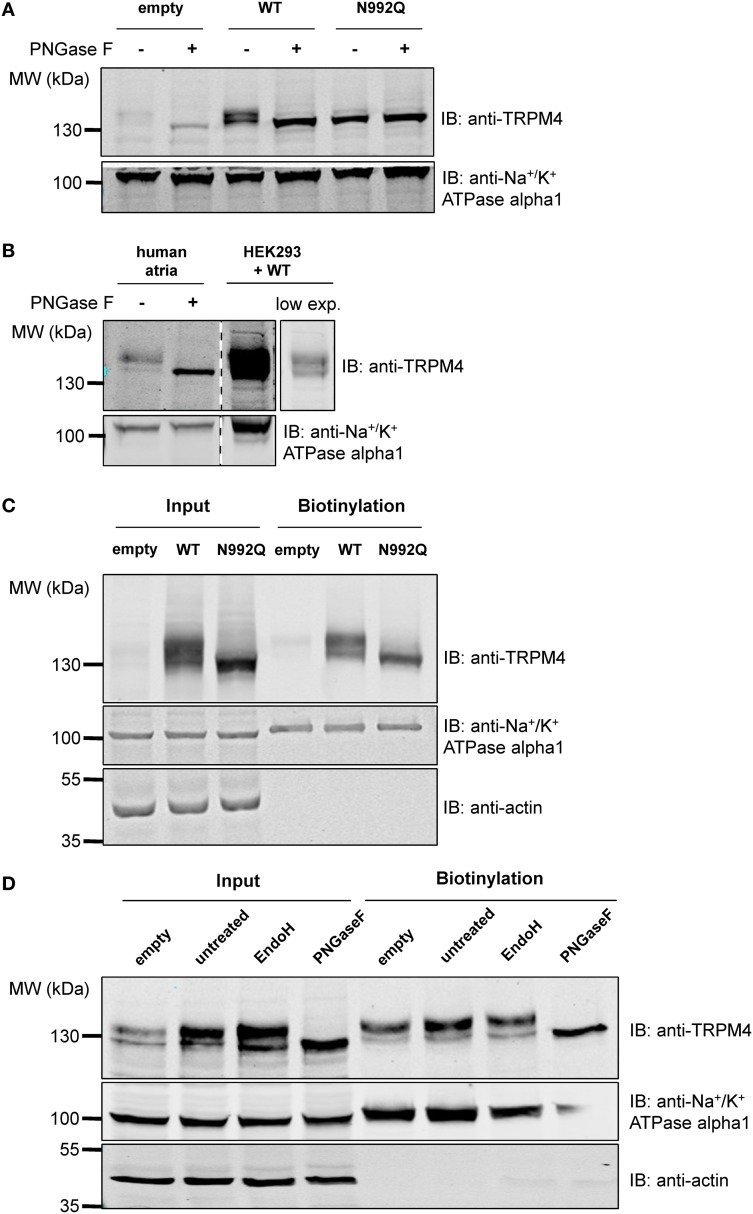
**Deglycosylation and cell surface biotinylation of TRPM4. (A)** Treatment with Peptide N-glycosidase F (PNGase F) demonstrates that the unglycosylated WT TRPM4 has a similar molecular weight to the TRPM4 N992Q mutant in HEK293 cells transiently transfected with WT or N992Q mutant. **(B)** Treatment of TRPM4 isolated from human cardiac atria with PNGase F shows that glycosylation is also found on TRPM4 from native tissues. Lane 4 is a lower exposure image of lane 3. All samples were run and blotted on the same membrane with rearrangement intended for clarity purposes. **(C)** Cell surface biotinylation of HEK293 cells transiently transfected with WT or TRPM4 N992Q mutant constructs shows expression of both WT and mutant at the total level and at the cell surface. **(D)** Treatment with Endo H only removes some of the glycosylation in the input fraction, but not in the biotinylated fraction.

As membrane proteins are often glycosylated and TRPM4 was migrating as a distinct doublet on SDS-PAGE gels, TRPM4 was then searched for the presence of an N-glycosylation consensus sequence Asn-Xaa-Ser/Thr (Xaa being any amino acid except Pro and Asp), using the publicly available programme NetNGlyc 1.0 Server (http://www.cbs.dtu.dk/services/NetNGlyc). A putative N-glycosylation motif, Asn-Cys-Ser at residue Asn^992^ (Figure 1A), located after the selectivity filter (981-EDMDVA-986) between segment 5 and 6 (Figure 1B), was detected. This consensus sequence is conserved in humans, mice, and rats (Figure 1A), while among other TRPM channels, only TRPM5 displays a similar signal at residue Asn^932^ (Figures 1A,B). To test the hypothesis that Asn^992^ is the TRPM4 glycosylation site, Asn was mutated into Gln (N992Q) to enable the investigation of the cell biology and functional roles of TRPM4 glycosylation. As illustrated in Figure 2, WT TRPM4 runs as a distinct doublet (Figure 2A) in Western blot experiments. The N992Q mutation resulted in a single band running faster than the lower band of the WT protein (compare lanes 3 and 5 in Figure 2A). This band was unaffected by treatment with Peptide-N-glycosidase F (PNGase F), which trims all N-linked glycosylation modifications (Figure 2A lane 6). Thus, this band was subsequently named *unglycosylable* TRPM4. Upon treatment with PNGase F, the two bands seen in the WT were also shifted into a single band running at the same height as the unglycosylable band (compare lanes 4 and 5 in Figure 2A). This observation led us to conclude that the upper and lower bands are fully- and core glycosylated TRPM4 fractions, respectively, and that Asn^992^ is the unique glycosylation site of TRPM4. The lanes where lysates of non-transfected HEK293 cells were loaded (*empty* in Figure 2A) show similar findings as the WT, albeit with much fainter signals. These bands are most likely endogenous TRPM4 expressed in HEK293 cells, as previously reported by our group (Amarouch et al., 2013). To further confirm that glycosylated TRPM4 exists *in vivo*, total protein from a piece of human cardiac atria was isolated and treated with PNGase F. As demonstrated in Figure 2B, endogenous cardiac TRPM4 ran as a distinct doublet (lane 1), which shifted into a single band (lane 2) running slightly lower than the core glycosylated band when treated with PNGase F. This result demonstrated that fully- and core glycosylated forms of TRPM4 are endogenously expressed in cardiac cells.

### Glycosylation is not essential for the expression of TRPM4 at the cell surface

To determine whether glycosylation is essential for TRPM4 transport and expression at the cell surface, cell surface biotinylation assays were performed. As shown in Figure 2C (lanes 3 and 6), TRPM4 N992Q mutant was well expressed both in the whole cell lysate and at the cell surface, despite being unglycosylated (Figure 2C). This experiment suggests that glycosylation is not essential for expression of TRPM4 at the cell surface.

### TRPM4 N-glycosylation types are different in the intracellular fraction and at the cell surface

In order to obtain more information about the N-glycosylation identity of TRPM4, TRPM4 was treated with endoglycosidase H (Endo H) in addition to PNGase F. While PNGase F trims all N-linked glycosylation (high mannose, hybrid and complex oligosaccharides) from the innermost GlcNAc (N-Acetylglucosamine) that is linked to an Asn residue, Endo H only trims high mannose and certain hybrid oligosaccharides, but not complex oligosaccharides (Maley et al., 1989). As illustrated in Figure 2D, PNGase F completely trims TRPM4 in total and biotinylated fractions (Figure 2D lanes 4 and 8). However, Endo H only trims a part of glycosylated TRPM4 in the input fraction (Figure 2D lane 3), and none in the biotinylated fraction (Figure 2D lane 7). This observation suggests that TRPM4 has a significant amount of complex oligosaccharides and a minor amount of high mannose and/or hybrid oligosaccharides in the total fraction where TRPM4 is mostly intracellular, while only complex oligosaccharides exist at the cell surface.

### The N992Q mutant decreases TRPM4 current density

**Figure 3:**
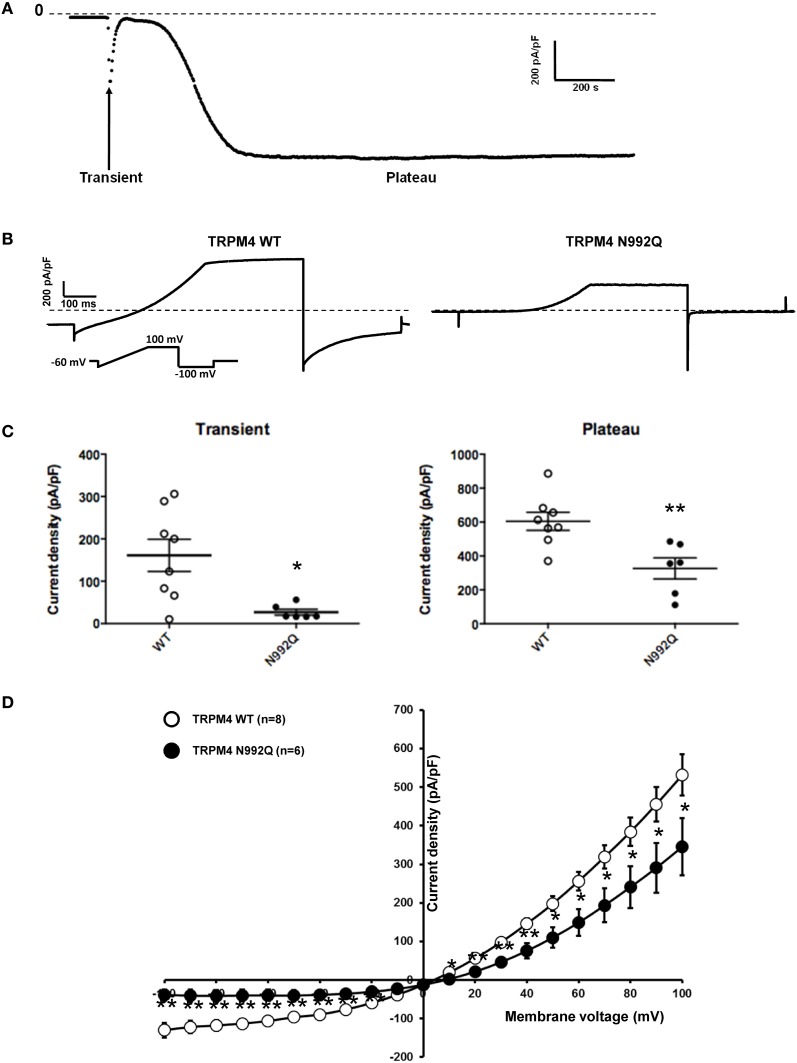
**Whole cell patch clamp recording of WT and TRPM4 N992Q mutant transiently transfected in HEK293 cells. (A)** Current density of TRPM4 over time shows a brief transient and a long persistent plateau phase. **(B)** Individual currents recorded using a ramp protocol depicted in the inset. **(C)** Quantification at +100 mV of WT and TRPM4 N992Q mutant current densities during the transient (left panel) and plateau (right panel) phases in dot plot graph shows a significant decrease in current densities in both phases compared to TRPM4 WT. **(D)** Current vs. voltage relationship shows a decreased current in TRPM4 N992Q mutant without major alterations of the voltage-dependence. Statistical test using two-tailed Student *T*-test ^*^P < 0.05, ^**^P < 0.01.

Amarouch et al. (2013) used the patch clamp technique in whole-cell configuration with HEK293 cells, to demonstrate that the TRPM4-mediated current recorded over time shows two distinct phases (Figure 3A). A transient phase appears quickly after the rupture of the membrane patch, and then a plateau phase appears 1–3 min later (Figure 3A). To investigate the consequences of the N992Q mutation on TRPM4 function, the transient and plateau current density were recorded at +100 mV and −100 mV using a ramp protocol (Figure 3B). The quantification of both groups (WT and N992Q) showed a statistically significant decrease in current density of both the transient and plateau phases (WT vs. N992Q transient: 161 ± 38 pA/pF *n* = 8 vs. 27 ± 7 pA/pF *n* = 6; plateau: 604 ± 53 pA/pF *n* = 8 vs. 327 ± 62 pA/pF *n* = 6) (Figures 3B,C). In addition, the deactivation kinetics of TRPM4 at the end of the step at +100 mV and −100 mV were faster with the N992Q mutant compared to WT (Figure 3B). Due to the extremely fast deactivation at −100 mV, this parameter was only quantified at +100 mV (WT 3749 ± 471 pA/s *n* = 9 vs. N992Q −90 ± 139 pA/s *n* = 8, *p* < 0.05). In parallel, the current-voltage (I-V) relationship, performed during the plateau phase, did not reveal any drastic alterations in the voltage-dependence of the mutant channel (Figure 3D).

### TRPM5 channel is N-linked glycosylated at Asn^932^

**Figure 4:**
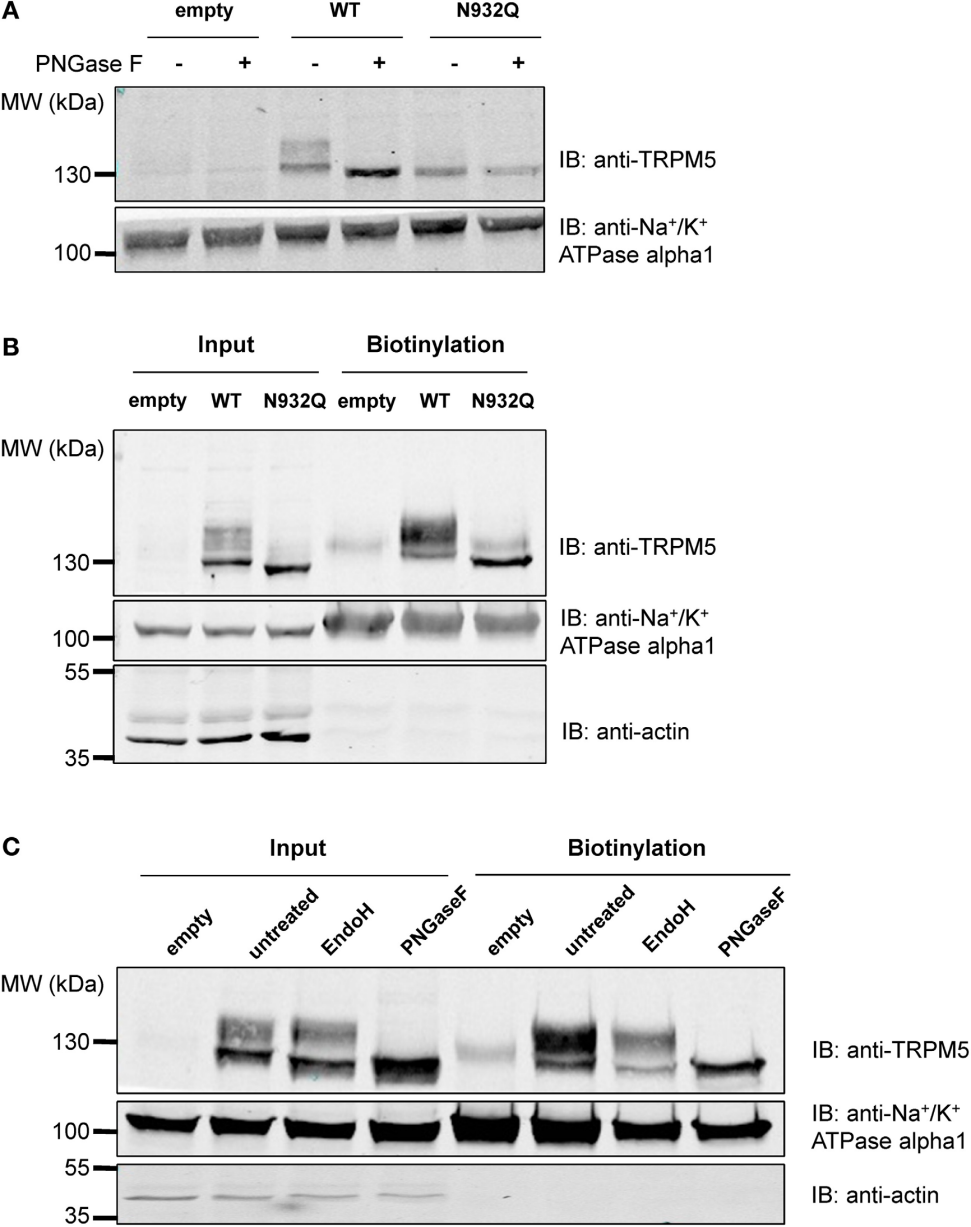
**Deglycosylation and biotinylation of TRPM5. (A)** Treatment with PNGase F demonstrates that unglycosylated WT TRPM5 has a similar molecular weight to the TRPM5 N932Q mutant. **(B)** Cell surface biotinylation of HEK293 cells transiently transfected with WT or TRPM5 N932Q mutant constructs shows expression of both WT and mutant at the total level and at the cell surface. **(C)** Treatment with Endo H removes some of the glycosylation in both input and biotinylated fractions.

Similar experiments were performed with TRPM5, since it is the closest relative of TRPM4 in the TRPM channel family and it has a similar N-glycosylation signal, Asn-Cys-Ser, at residue Asn^932^ (Figure 1A). To test the hypothesis that Asn^932^ is where the glycoside residues link to TRPM5, Asn was mutated into Gln (N932Q). Using the same SDS-PAGE running conditions as for TRPM4, a lower band and a smeary upper band, presumably the core- and fully glycosylated bands, respectively, were observed with WT TRPM5 (Figure 4A lane 3). At the same time, the TRPM5 N932Q mutant appeared as a single band running faster than the lower band of the WT (Figure 4A lane 5), and this was concluded to be the unglycosylable band. When treated with PNGase F, the WT bands shifted into a single band (Figure 4A lane 4) running at the same height as the mutant band. This result confirms that Asn^932^ is the unique N-glycosylation site of TRPM5, and that the upper and lower bands are the fully- and core glycosylated bands of TRPM5, respectively.

### Glycosylation is not essential for the expression of TRPM5 at the plasma membrane

After concluding that glycosylation of TRPM4 is not essential for its expression at the cell surface, tests were performed to determine if this was also the case for TRPM5. Despite being unglycosylated, the TRPM5 N932Q mutant was well expressed and could be found in both the total fraction as well as the cell surface (Figure 4B lanes 3 and 6). This suggests that glycosylation is not essential for TRPM5 expression at the cell surface. The faint upper band observed in lanes 4 and 6 of Figure 4B may represent endogenous TRPM5 expressed in HEK293 cells.

### TRPM5 N-glycosylation types are similar in the intracellular fraction and at the cell surface

To learn more about the glycosylation identity of TRPM5, TRPM5 was treated with Endo H in addition to PNGase F, as was done for TRPM4. While PNGase F completely trimmed TRPM5 (Figure 4C lanes 4 and 8), Endo H only trimmed a small fraction of TRPM5 (Figure 4C lanes 3 and 7). Since no differences in the patterns between total and biotinylated fractions were observed, TRPM5 was concluded to have a majority of complex oligosaccharides with some minor amounts of high mannose and hybrid oligosaccharides in both fractions.

### TRPM5 current density is decreased with the N932Q mutant

**Figure 5:**
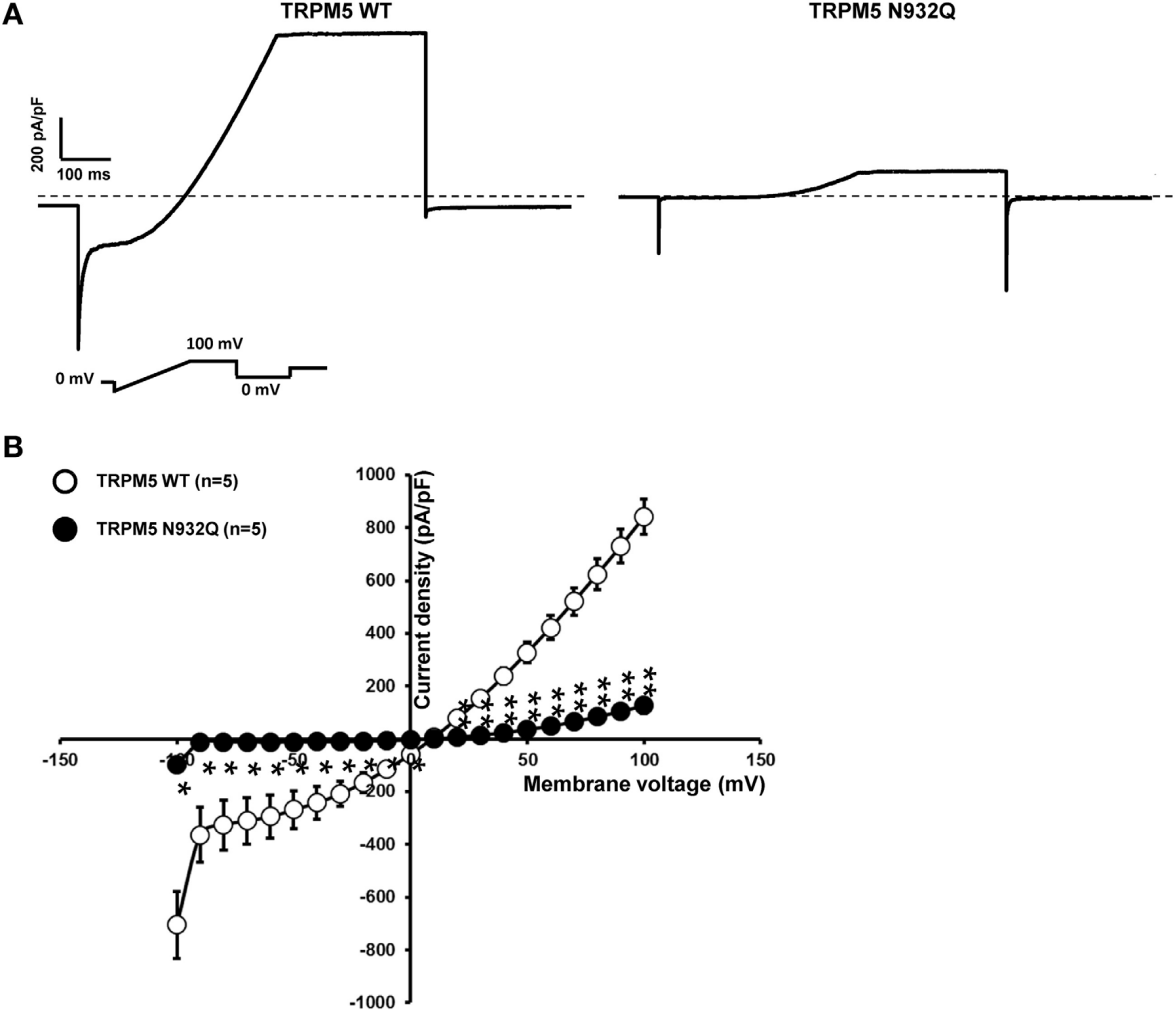
**Whole cell patch clamp recording of WT and TRPM5 N932Q mutant transiently transfected in HEK293 cells. (A)** Individual currents recorded using a ramp protocol depicted in the inset. **(B)** Current vs. voltage relationship shows a decreased current in TRPM5 N932Q mutant without major alterations of the biophysical properties. Statistical test using two-tailed Student *T*-test ^*^P < 0.05, ^**^P < 0.01.

Functional experiments using the similar patch clamp technique as was done for TRPM4 were performed. In the case of TRPM5, only one phase was observed after rupture of the membrane patch. The recordings revealed a statistically significant decrease in the current density at +100 mV for the N932Q mutant compared to WT (WT vs. N932Q: 848 ± 55 pA/pF *n* = 5 vs. 136 ± 26 pA/pF *n* = 5) (Figures 5A,B). No major alterations in the biophysical properties were observed (Figure 5B).

### Tunicamycin treatment increases TRPM4 current density

**Figure 6:**
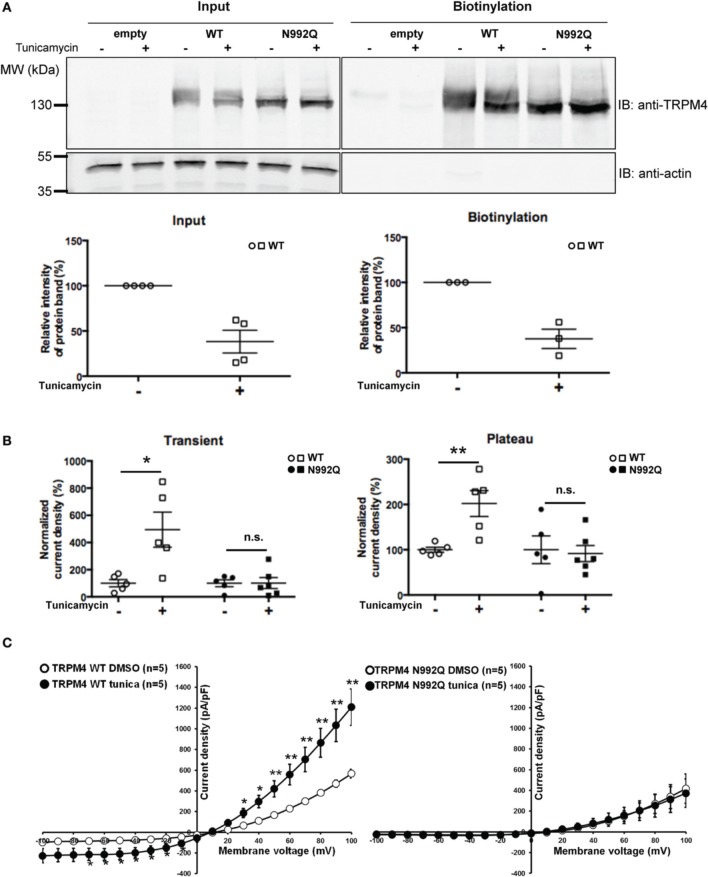
**Effect of tunicamycin treatment on WT and TRPM4 N992Q mutants. (A)** Cell surface biotinylation of HEK293 cells transiently transfected with WT or TRPM4 N992Q mutant constructs and pretreated with tunicamycin (10 μg/ml; 19 h) demonstrates a reduction of the glycosylated bands of TRPM4 following the treatment. Western blots shown in upper panels with quantification in lower panels (treated 38 ± 13% vs. untreated 100 ± 00% *n* = 4 in the input fraction; treated 38 ± 11% vs. untreated 100 ± 00% *n* = 3 in the biotinylated fraction; *p* < 0.05) **(B)** Quantification in dot plot graph of WT and N992Q mutant current densities at +100 mV shows a significant increase in the transient and plateau current densities after tunicamycin treatment (10 μg/ml; 19 h) only with the WT (transient: untreated 123 ± 37 pA/pF *n* = 5 vs. treated 610 ± 178 pA/pF *n* = 5; *p* < 0.05; plateau: untreated 657 ± 41 pA/pF *n* = 5 vs. treated 1327 ± 210 pA/pF *n* = 5; *p* < 0.05), but not with the N992Q mutant (transient: untreated 23 ± 6 pA/pF *n* = 5 vs. treated 23 ± 10 pA/pF *n* = 6; plateau: untreated 426 ± 147 pA/pF *n* = 5 vs. treated 391 ± 83 pA/pF *n* = 6). Untreated: vehicle (DMSO). **(C)** Current vs. voltage relationship shows an increase of current in TRPM4 WT condition after tunicamycin treatment, without major alterations in voltage dependence. Statistical test using two-tailed Student *T*-test ^*^P < 0.05, ^**^P < 0.01.

Since the TRPM4 N992Q mutation reduced its current without any striking biophysical alterations, it remained to be determined whether the effect was primarily dependent on the Asn to Gln mutation itself or on the consequence of the mutation, i.e., the lack of TRPM4 glycosylation. Thus, HEK293 cells transiently expressing either WT TRPM4 or N992Q mutant were treated with tunicamycin, an inhibitor of N-linked glycan synthesis. Tunicamycin treatment at 10 μg/ml for 19 h decreased the upper band by approximately 60%, as shown in Figure 6A. Somewhat unexpectedly, this treatment led to a statistically significant increase in both the transient and plateau current densities of TRPM4 WT (normalized untreated vs. treated transient; 100 ± 27% *n* = 5 vs. 494 ± 129% *n* = 5 and plateau; 100 ± 6% *n* = 5 vs. 202 ± 29% *n* = 5) (Figure 6B) without modifying the I-V relationship (Figure 6C) or the deactivation kinetics at +100 mV (WT DMSO 4855 ± 379 pA/s *n* = 5 vs. WT tunicamycin 5265 ± 572 pA/s *n* = 5, *p* > 0.05). Importantly, the same treatment on HEK293 cells expressing TRPM4 N992Q neither altered the current density recorded during both phases (untreated vs. treated transient; 100 ± 26% *n* = 5 vs. 102 ± 40% *n* = 6 and plateau; 100 ± 31% *n* = 5 vs. 92 ± 18% *n* = 6) nor the I-V relationship (Figures 6B,C). This suggests that the observed current density increase with TRPM4 WT under tunicamycin treatment is dependent on glycosylation of Asn^992^ (Figures 6A,B) and indicates that the N992Q-mediated current decrease depends on the mutation itself.

## Discussion

The main finding of this study is that in heterologous expression systems TRPM4 and TRPM5 are N-linked glycosylated, while TRPM4 was also found to be glycosylated in cardiac tissues. Furthermore, it was observed that this N-glycosylation occurs (1) via a unique site located in the pore-forming region, (2) may not play a significant role in trafficking to the plasma membrane, (3) can be of different types, and (4) is involved in modulating the current density at the cell surface.

### Glycosylation of TRPM4/5 channels

The pore region of the TRP channel, known to harbor the selectivity filter, may appear to be an unusual site for glycosylation. Several recent reports, however, have shown that TRPV1 and TRPV4 also have a unique N-glycosylation site in the same domain (Xu et al., 2006; Veldhuis et al., 2012). Furthermore, as cited by Cohen (2006), TRPV2 and TRPV3 are predicted to have N-glycosylation sites in the same region. Very recently, Woo et al. (2013a) investigated the site and the role of N-linked glycosylation of the mouse TRPM4. They showed biochemical evidence for TRPM4 glycosylation in native rat tissues, suggesting that this post-translational modification likely plays an important role *in vivo*. In the present work, we obtained evidence that fully- and core glycosylated forms of TRPM4 are expressed in human cardiac atrial tissue.

### Forward trafficking and glycosylation

N-linked protein glycosylation has been shown to be crucial for the trafficking of several ion channels toward the plasma membrane, i.e., Kv11.1 (hERG channel) (Petrecca et al., 1999) and Shaker channels (Khanna et al., 2001). In contrast, despite not having glycosylation, mutant TRPM4/5 and WT TRPM4/5 from tunicamycin-treated cells were well expressed at the plasma membrane, suggesting that N-glycosylation may not play an essential role in the forward trafficking. Woo and co-workers recently came to a similar conclusion (Woo et al., 2013a). Others have also reported that glycosylation is not essential for the forward trafficking of membrane proteins, such as TRPV5 (Leunissen et al., 2013), organic solute transporter alpha (Soroka et al., 2008) and proline-rich membrane anchor (Chan et al., 2012). In addition to the regulation of forward trafficking, N-glycosylation most likely has other important cellular functions, such as cell adhesion, self/non-self-recognition, receptor activation and endocytosis (Ohtsubo and Marth, 2006).

### Complex oligosaccharides and their role in TRPM4/5 regulation

As shown in Figure 2C, we observed that TRPM4 can have different types of N-glycosylation in the intracellular fraction (with a significant amount of complex oligosaccharides and a minor amount of high mannose and/or hybrid oligosaccharides), and at the cell surface (exclusively with complex oligosaccharides). Woo et al. (2013a) also showed analogous results for the total protein fraction, although they only mentioned the presence of high mannose in TRPM4. The significant amount of complex oligosaccharides found in the total fraction (intracellular), which are a mix of cytoplasm, endoplasmic reticulum (ER), Golgi apparatus, plasma membrane might come from the bulk TRPM4 Golgi and/or downstream compartments where TRPM4 is mostly glycosylated with complex oligosaccharides. Nonetheless, we cannot rule out the possibility that ER membranes were not efficiently disrupted to release ER-bound TRPM4 which is most likely glycosylated with high mannose and/or oligosaccharides. Such a difference of glycosylation types in the two fractions was, however, not observed with TRPM5, despite the fact that the site of glycosylation is found in a homologous domain. It is also interesting to note that most membrane proteins are glycosylated with complex oligosaccharides, but not with high mannose and/or hybrid oligosaccharides at the cell surface. However, we observed that TRPM5 is glycosylated with a significant amount of complex oligosaccharides and a minor amount of high mannose and hybrid oligosaccharides both intracellularly and at the cell surface. Others groups (Tulsiani et al., 1992; Kuo et al., 1996; Meschi et al., 2005), however, have found high mannose and/or hybrid oligosaccharides on plasma membrane-bound proteins. Taking these observations into account, it is possible to find TRPM5 glycosylated with high mannose and/or hybrid oligosaccharides at the cell surface.

### Unglycosylable TRPM4/5 mutants

In the present study, the consequences of the Asn to Gln mutations on the function of the TRPM4/5 channels were further investigated. The unglycosylable TRPM4/5 mutants displayed strikingly decreased current amplitudes compared to their respective controls, despite the fact that their total protein expression at the cell surface was not altered. This finding suggests that N-glycosylation of TRPM4/5 has a stronger impact on the channel's function than on its expression at the plasma membrane. Nevertheless, the observed discrepancy between the biochemistry and functional findings is puzzling. It may be that the decreased TRPM4/5 current densities are caused by the mutation itself, rather than by the fact that the channels are not glycosylated. Woo et al. (2013a) showed that the single channel conductance of the mouse TRPM4 channel, which harbors the homologous Asn to Gln mutation, was not altered. It may be, however, that the observed whole-cell current reduction could be due to alterations of open probability that were not measured by the authors.

### Tunicamycin treatment of WT and unglycosylable TRPM4

Since the N-glycoslyation signal (Asn^992^) of TRPM4 is close to its selectivity filter, the observed current decrease with the N992Q mutant could be due to either an alteration of TRPM4 gating mediated directly by the mutation itself or as a consequence of absence of glycosylation. To discriminate between these two possibilities, TRPM4 currents were recorded after treating the cells with the glycosylation inhibitor, tunicamycin, in order to determine whether the reduction of the TRPM4 N992Q current amplitude was mainly a consequence of the lack of glycosylation. Somewhat unexpectedly, following this treatment both plateau and transient TRPM4-mediated current densities were greater, without any evidence of an increase in the number of channels expressed at the plasma membrane. Importantly, tunicamycin had no effect on the unglycosylable TRPM4 N992Q mutant. This finding suggests (1) that the tunicamycin-mediated current increase was due to the deglycosylation of the channel rather than the altered glycosylation of other proteins, and (2) that the N992Q-induced reduction of current was caused by the mutation itself rather than its effect on glycosylation. As a consequence, these results indicate that the N992Q mutant is not the most suitable model for the investigation of the functional roles of TRPM4 glycosylation. Analogous to the aforementioned reduction in the current of the unglycosylable mutants, the intensity of the total TRPM4 protein signal at the cell surface (both fully- and core glycosylated TRPM4) was not increased after tunicamycin treatment, while the current density was augmented. These discrepant observations may be due to limitations of the technique. As observed in Figure 6A, when compared to the untreated condition, the tunicamycin treatment decreased the fully glycosylated band of TRPM4 WT and increased the intensity of the core N-glycosylated band. A similar observation was also made by Leunissen et al. (2013) after Endo F treatment of WT TRPV5. It is possible that under these conditions the quantification of the TRPM4 signal is biased because the affinity of the antibody may vary depending on the N-glycosylation pattern, as has been previously suggested (Reynard et al., 2004). Another explanation of this discrepancy could be that the TRPM4 single channel properties are altered after tunicamycin treatment.

## Conclusions

Altogether, the results presented in this study demonstrate that N-linked glycosylation at a unique site on both TRPM4 and TRPM5 is involved in the regulation of channel activity. Additionally, these results also suggest that the region surrounding the selectivity filter (Nilius et al., 2005a) is involved in the channel's function. Further experiments are needed to better understand the role of this unique glycosylation site in modulating the biophysical properties of both TRPM4 and TRPM5 channels. In summary, we have mapped the N-glycosylation site of TRPM4/5 and have identified the types of N-glycosylation displayed by both channels. More importantly, we were able to demonstrate that glycosylation impacts channel function but not its surface expression.

## Author contributions

Ninda Syam designed, performed experiments and wrote the manuscript. Jean-Sébastien Rougier designed, performed experiments and wrote the manuscript. Hugues Abriel designed and supervised the project and wrote the manuscript.

### Conflict of interest statement

The authors declare that the research was conducted in the absence of any commercial or financial relationships that could be construed as a potential conflict of interest.
